# Switching single chain magnet behavior *via* photoinduced bidirectional metal-to-metal charge transfer†
†Electronic supplementary information (ESI) available: Synthesis and physical measurement details. Crystal data in CIF format and additional figures (Fig. S1–S15). CCDC 1528877. For ESI and crystallographic data in CIF or other electronic format see DOI: 10.1039/c7sc03401f


**DOI:** 10.1039/c7sc03401f

**Published:** 2017-10-30

**Authors:** Wenjing Jiang, Chengqi Jiao, Yinshan Meng, Liang Zhao, Qiang Liu, Tao Liu

**Affiliations:** a State Key Laboratory of Fine Chemicals , Dalian University of Technology , 2 Linggong Rd. , 116024 , Dalian , China . Email: liutao@dlut.edu.cn

## Abstract

The preparation of single-chain magnets (SCMs) with photo-switchable bistable states is essential for the development of high-density photo-recording devices. However, the reversible switching of the SCM behavior upon light irradiation is a formidable challenge. Here we report a well-isolated double zigzag chain {[Fe(bpy)(CN)_4_]_2_[Co(phpy)_2_]}·2H_2_O (bpy = 2,2′-bipyridine, phpy = 4-phenylpyridine), which exhibits reversible redox reactions with interconversion between FeIIILS(μ-CN)CoIIHS(μ-NC)FeIIILS (LS = low-spin, HS = high-spin) and FeIIILS(μ-CN)CoIIILS(μ-NC)FeIILS linkages under alternating irradiation with 808 and 532 nm lasers. The bidirectional photo-induced metal-to-metal charge transfer results in significant changes of anisotropy and intrachain magnetic interactions, reversibly switching the SCM behavior. The on-switching SCM behavior driven by light irradiation at 808 nm could be reversibly switched off by irradiation at 532 nm. The results provide an additional and independent way to control the bistable states of SCMs by switching in the 0 → 1 → 0 sequence, with potential applications in high density storage and molecular switches.

## Abstract 2

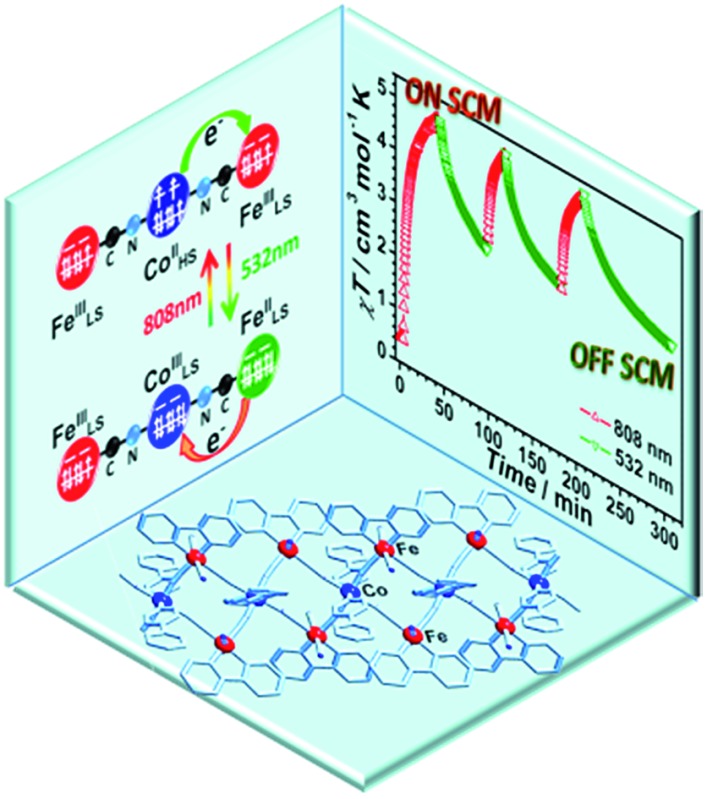
The on/off-switching of single-chain magnetic behavior tuned through bidirectional light irradiation.

## Introduction

Single chain magnets (SCMs) possess bistable states (relative to binary 0 and 1) and slow magnetic relaxation,^1^–^3^ rendering them promising candidates for high-density information storage, quantum computing and spintronic devices.^4^–^7^ Thus, SCMs have attracted considerable attention and been widely studied in chemistry, physics and materials science. Various SCM systems have been developed,^8^–^21^ wherein particular interest is paid to designing switchable SCMs that are responsive to external stimuli, such as guest molecules, pressure, light and heat, *etc.*^15^–^21^ The unique magnetic properties of SCMs stem from large single-ion anisotropy, strong intrachain interactions and weak interchain interactions. When externally stimulated changes in single-ion anisotropy and strong intrachain interactions are embedded, it becomes possible to reversibly generate or cancel the bistable states of SCMs. Such switchable SCMs provide an additional and independent way to control the magnetic states by switching in the 0 → 1 → 0 sequence, which is supposed to increase the performance of currently used standard molecular model device-systems that exploit electronic read-out and transverse field techniques.^6^,^7^ Thus, the swift and reversible production and elimination of the SCM behavior is of great importance in the face of future technological demand for data storage and processing.

Light-induced switchable nanomagnets provide the opportunity to achieve this goal.^17^–^26^ This is because the light-induced process usually occurs on the picosecond scale,^27^ enabling a rapid response of functional signals upon light irradiation. Moreover, not only is light convenient to be turned on/off and switched between different wavelengths, but it also offers matched energy for the interconversion of different electronic states. Benefiting from these, the light-driven SCMs were achieved in cyanide-bridged Fe–Co assemblies,^17^–^19^ where the SCM properties in the sequence of off → on were induced by the light-induced metal-to-metal charge transfers (MMCTs)^28^–^38^ or excited spin-state trapping (LIESST) processes.^20^ However, examples of light-driven SCMs are still limited due to the difficulty in combining SCM behavior with the photo-responsive property. Moreover, the manipulation of light-driven SCMs is all unidirectional for the reported examples, wherein the SCM behavior can be activated *via* the light irradiation process but deactivated only *via* a time consuming thermal relaxation process upon heating.^17^–^20^ No reversible photo-switchable SCMs have been reported, and the development of such systems remains a big challenge to fulfil the reversible control of magnetic states by lights analogous to the switch in the off → on → off sequences.

In this study, we seek to design a system that displays the on/off-switching SCM properties tuned through light irradiation. There are two challenges for the realization of this goal. One crucial problem is the introduction of photo-responsive units in which the electronic states of the constituent metal ions can be reversibly photo-switched. The other is to suitably assemble the photo-sensitive units into a magnetically well-isolated 1D chain associated with large uniaxial magnetic anisotropy and strong intrachain magnetic interactions. An applicable example is the Fe/Co Prussian blue analogues,^28^,^39^–^41^ as the bidirectional light-induced MMCTs were discovered in several Fe_2_Co_2_ square compounds.^40^,^41^ This discovery inspired us to introduce such a reversibly photo-switchable Fe_2_Co_2_ square into a double zigzag chain, wherein SCM behavior^42^–^45^ and photo-driven SCMs have been reported.^17^,^18^ Herein, we selected Li[Fe^III^(bpy)(CN)_4_] (bpy = 2,2′-bipyridine) as the building block to react with Co^II^ ions and 4-phenylpyridine (phpy), forming a well-isolated 1D complex {[Fe(bpy)(CN)_4_]_2_Co(phpy)_2_}·2H_2_O (**1**). The cyanide bridges enabled the linkage of the Fe^III^ and Co^II^ ions in a 1D alignment and provided super-exchange pathways for strong magnetic interactions. Phpy was adopted as the auxiliary ligand to adjust the coordination sphere of the Co^II^ sites, providing an appropriate ligand field and redox potential for the interconversion between the paramagnetic FeIIILS(μ-CN)CoIIHS (HS = high-spin, LS = low-spin) and diamagnetic FeIILS(μ-CN)CoIIILS linkages. The HS Co^II^ ion (*S* = 3/2) is paramagnetic and is supposed to have strong anisotropy,^42^–^46^ whereas the LS Co^III^ ion is diamagnetic (*S* = 0) and has no anisotropy, offering the potential for photo-induced anisotropy.^17^–^19^,^26^,^47^–^49^ The interconversion between the FeIIILS(μ-CN)CoIIHS and FeIILS(μ-CN)CoIIILS linkages will switch the anisotropy and intrachain magnetic interactions that are essential for the SCM behavior, providing the possibility of switchable SCM behavior *via* a photo-induced bidirectional MMCT upon light irradiation. As a result, **1** represents the first example showing SCM behavior which could be switched on/off *via* bidirectional MMCT under alternating irradiation with 808 nm and 532 nm lasers.

## Results and discussion

### X-ray crystal structure

**Fig. 1:**
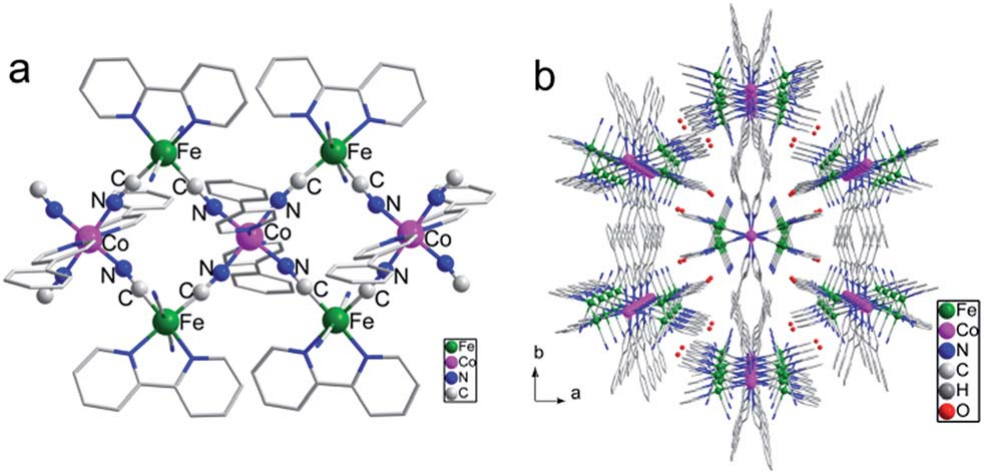
(a) Side view of the 1D chain. (b) Packing diagram of the 1D chains along the *c* axis. The hydrogen atoms are omitted for clarity. Fe, green; Co, pink; C, gray; N, blue.

The crystal of **1** suitable for X-ray diffraction was obtained by the diffusion method from the methanol solution of Li[Fe(bipy)(CN)_4_]·H_2_O and phpy to the water solution of Co(ClO_4_)_2_·6H_2_O. A single-crystal X-ray diffraction experiment revealed that **1** crystalized in the monoclinic space group *C*2/*c*. The repeating unit comprised {[Fe(bpy)(CN)_4_]_2_Co(phpy)_2_} and the units were connected with each other *via* cyanide bridges, forming an infinite double zigzag chain along the *c* axis (Fig. 1a). Uncoordinated water molecules were located between the chains (Fig. 1b). Within the repeating unit, the cobalt ion was coordinated to four cyanide nitrogen atoms from two adjacent [Fe(bpy)(CN)_4_]^–^ entities and two nitrogen atoms from the monodentate phpy ligands, forming an elongated octahedral environment. At 220 K, the Co–N_cyanide_ and Co–N_phpy_ distances were 1.885(3)–1.892(3) and 1.975(3) Å, respectively, which are characteristic of the LS Co^III^ ions. The Fe–C and Fe–N bond distances were 1.882(3)–1.955(4) and 1.981(3)–1.985(3) Å, respectively. These structural characteristics and charge compensations suggested the existence of FeIIILS(μ-CN)CoIIILS(μ-NC)FeIILS linkages in **1**. However, due to the inversion symmetry requirement, the FeIIILS and FeIILS can not be distinguished and may be randomly located in the Fe_2_Co entity.^17^,^18^ Between the repeating units, the planes of the triangular Fe_2_Co units were arranged in an interlaced pattern with the dihedral angle of 34.2°. The shortest interchain Co···Co distance was 15.099 Å.

Such a large distance would diminish the interchain magnetic interactions, meeting the requirements of isolation for SCM behavior well.

### Mössbauer spectra analysis

^57^Fe Mössbauer spectroscopy is very powerful for determining the electronic states of the Fe centers. As shown in Fig. S1 in the ESI,† two quadrupole doublets were observed at 78 K. One is characteristic of the LS Fe^III^ species with Mössbauer parameters of *δ* (isomer shift) = –0.03 and Δ*E*_Q_ (quadrupole splitting) = 1.69 mm s^–1^, and another can be attributed to the LS Fe^II^ species with *δ* = 0.07 and Δ*E*_Q_ = 0.77 mm s^–1^. The Fe^III^/Fe^II^ peak area ratio was 0.51/0.49, confirming the LS state of **1** with the FeIIILS(μ-CN)CoIIILS(μ-NC)FeIILS linkages.

### Magnetic characterization

Magnetic measurements further confirmed the LS state of **1**. The temperature dependence of the magnetic susceptibilities was measured over the 2–300 K temperature range in the heating mode (Fig. S2a, ESI†). As the temperature increased, the *χT* values for each Fe_2_Co remained close to 0.51 cm^3^ K mol^–1^, corresponding to one LS Fe^III^ ion. The results suggested that the electronic state of the intrachain is described as FeIIILSCoIIILSFeIILS. The field-dependent magnetization at 1.8 K increased slowly to 1.01 Nβ at 50 kOe, consistent with the value expected for one LS Fe^III^ ion (Fig. S2b, ESI†). The alternating current (ac) magnetic susceptibilities were studied as a function of both temperature and frequency. No temperature and frequency dependence were observed for both in-phase (*χ*′) and out-of-phase (*χ*′′) components, corresponding to the pure paramagnetic behavior of the FeIIILSCoIIILSFeIILS unit (Fig. S2c, ESI†).

### On-switching SCM (808 nm)

**Fig. 2:**
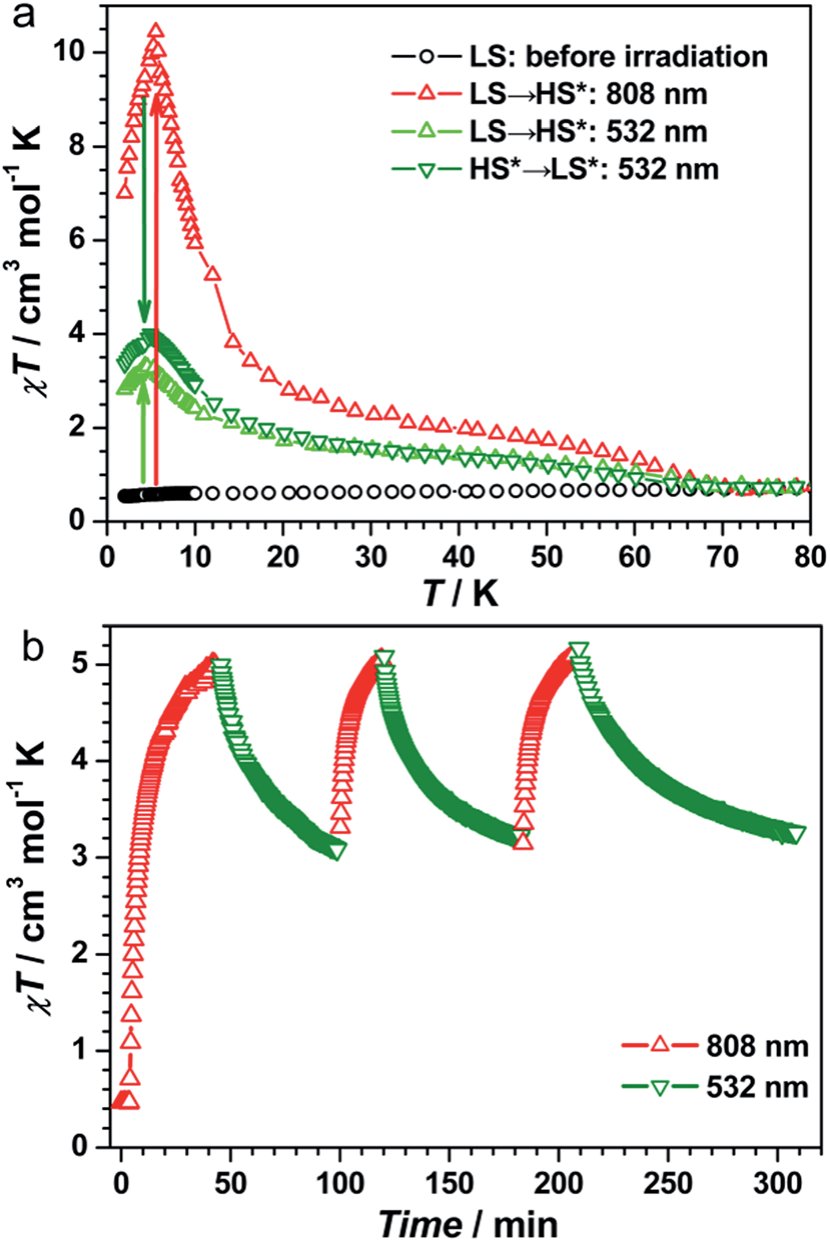
(a) Plots of *χT vs.* temperature for **1** before irradiation (LS) and after irradiation at 808 or 532 nm (LS → HS*), and the 808 nm-induced metastable state irradiated at 532 nm (HS* → LS*). (b) Plots of *χT vs.* time under cycles of successive irradiation at 808 nm and 532 nm at 10 K for **1**.

**Fig. 3:**
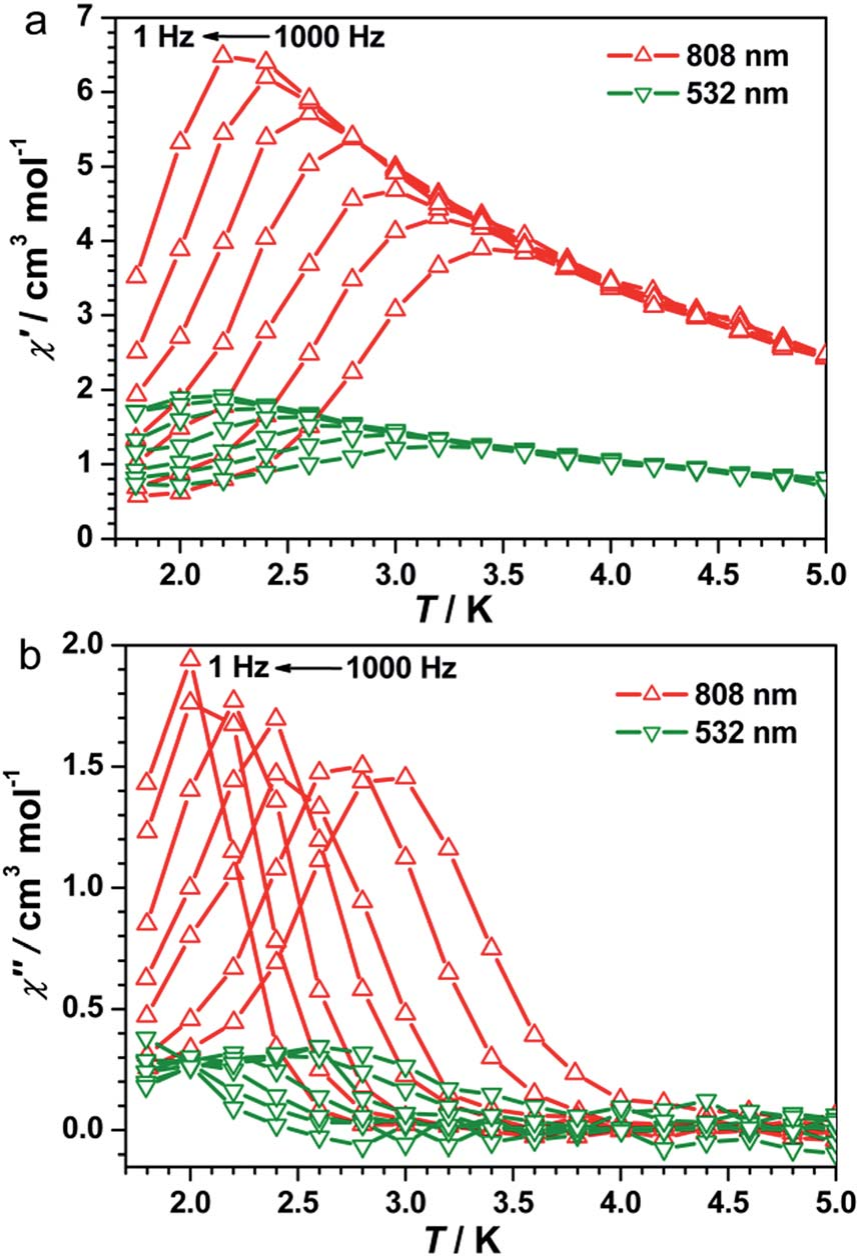
Temperature dependence of the in-phase (a) and out-of-phase (b) components of ac magnetic susceptibility after irradiation at 808 nm and 532 nm in a zero dc field at various ac frequencies and with a 3.5 Oe ac field.

The UV-vis spectra of **1** showed a broad band at *λ* = 750 nm at 298 K (Fig. S3, ESI†), which can be assigned to the Fe^II^ → Co^III^ intervalence charge transfer (IVCT).^35^,^40^,^41^ Therefore, an 808 nm laser was used to irradiate **1** at 10 K to explore the possibility of photoinduced transformation from FeIIILS(μ-CN)CoIIILS(μ-NC)FeIILS linkages to FeIIILS(μ-CN)CoIILS(μ-NC)FeIIILS linkages. The time-dependent *χT* values increased significantly from 0.47 cm^3^ mol^–1^ K before irradiation, reaching a saturation value of 5.62 cm^3^ mol^–1^ K after 9000 s under irradiation at 808 nm (Fig. S4, ESI†). This result indicated the generation of the metastable paramagnetic FeIIILS(μ-CN)CoIIHS(μ-NC)FeIIILS units (HS* state) transforming from the diamagnetic FeIILS(μ-CN)CoIIILS(μ-NC)FeIIILS units (LS state). The temperature-dependent *χT* product was then collected for the photo-induced HS* state from 1.8 to 120 K (Fig. 2a). The *χT* values increased steeply to a sharp maximum of 10.5 cm^3^ mol^–1^ K at 5.0 K before the HS* phase relaxed back to the LS phase on thermal treatment up to 80 K. The maximum *χT* value after irradiation indicated the occurrence of intrachain ferromagnetic interactions between FeIIILS (*S* = 1/2) and photo-induced CoIIHS (*S* = 3/2).^17^–^19^,^26^ Simultaneously, the field-dependent magnetization recorded after irradiation at 1.8 K showed a steep increase below 5 kOe, followed by an almost linear increase to 2.03 Nβ at 50 kOe (Fig. S5, ESI†). This evolution of the field-dependent magnetization replicated the production of ferromagnetic interactions in the photo-induced HS* chain. Considering the negative anisotropy of the Co^II^ ion and the intrachain ferromagnetic interactions transmitted *via* the cyanide bridges, the SCM behavior was expected.^17^–^19^,^42^–^45^ Alternating current (ac) magnetic susceptibility measurements were then carried out as a function of both temperature and frequency to investigate the magnetization dynamics of the photo-generated HS* phase. Both the in-phase (*χ*′) and the out-of-phase (*χ*′′) signals showed strong frequency dependence (Fig. 3 and S6a, ESI†). The shift of the peak temperature (*T*_p_) was characterized by the parameter *Φ* = (Δ*T*_p_/*T*_p_)/Δ(log *f*) = 0.13, within the expected range for SCMs, thus excluding the possibility of spin-glass behavior and magnetic ordering.

Detailed parameters were estimated and fitted by the Arrhenius law on the basis of the peak values of *χ*′′ (Fig. S7, ESI†), giving a pre-exponential factor of *τ*_0_ = 2.48 × 10^–10^ s and the relaxation energy barrier of *Δ*/*k*_B_ = 40.21 K. The value of *τ*_0_ provides a quantitative estimation of the attempt time of relaxation from the chain bath and the obtained value is in good agreement with those reported for other photo-induced SCMs.^17^–^20^ A semicircular Cole–Cole diagram was constructed from the variable-frequency data collected at 2.7 K and was fitted with a generalized Debye model (Fig. S8, ESI†), yielding an *α* value of 0.33 that is similar to those obtained for other photo-induced SCMs.^17^–^20^ The one-dimensional behavior after irradiation at 808 nm was further checked with ac data at 1 Hz in a zero dc field following the correlation length that is proportional to the *χ*′*T* product in any 1D classical system. The ln(*χ*′*T*) *vs. T*^–1^ plot exhibited a clear linear region between 7 and 25 K (Fig. S9a, ESI†), supporting an anisotropic Ising-like 1D behavior.^50^–^52^ The linear region was fitted according to the equation *χT* = *C*_eff_ exp(*Δ*_ξ_/*k*_B_*T*), where *C*_eff_ is the effective Curie constant and *Δ*_ξ_ is the energy of creating a domain wall along the chain, giving *Δ*_ξ_/*k*_B_ = 12.08 K and *C*_eff_ = 1.71 cm^3^ mol^–1^ K. Below 4.0 K, ln(*χ*′*T*) reached saturation owing to the finite-size effects with [*χ*′*T*]_max_ = 15.35 cm^3^ mol^–1^ K which was equal to *nC*_eff_, where *n* was the unit number in the chain. Hence, *n* could be estimated to be approximately 9.0, which is comparable to the values for reported SCMs.^53^ Therefore, all analyses together confirmed that compound **1** became a photo-driven SCM after irradiation at 808 nm.

### Off-switching SCM (532 nm)

To exploit the photo-reversibility of the photo-driven SCM behavior, the photo-induced metastable HS* state was irradiated at 405, 532, 473, and 671 nm followed by measuring the magnetic susceptibilities.^40^,^41^ The most significant effect was observed at 532 nm, where *χT* values decreased obviously and dropped to the minimum value after 10 000 s, from 5.62 to 2.85 cm^3^ mol^–1^ K (Fig. S4, ESI†). This phenomenon could be ascribed to the partial elimination of the HS* FeIIILS–CoIIHS metastable state under irradiation at 532 nm, causing partial recovery of the diamagnetic FeIILS–CoIIILS pairs. After deactivating the 532 nm laser, the temperature-dependent *χT* values were measured from 1.8 to 120 K. The maximum value of *χT* decreased to 4.00 cm^3^ mol^–1^ K at 5 K. This value was much smaller than that obtained after 808 nm irradiation (Fig. 2a). The field-dependent magnetization decreased to 1.58 Nβ at 50 kOe (Fig. S5, ESI†). Different to the dynamic behavior of the HS* state, the LS* state showed a poor frequency dependence behavior and weak intensity in *χ*′ and *χ*′′ signals (Fig. 3 and S6b, ESI†). It was probably because most of the effectively coupled chains have been destroyed, therefore the SCM behavior is lost. As shown in Fig. S9b,† the typical linear region for the 1D chains disappeared, definitely confirming that the SCM behavior driven by irradiation at 808 nm was broken up by the 532 nm laser. Due to the incomplete conversion from the HS* state to LS* state, the remnant frequency dependence may possibly result from the segments of coupled spins.

As found in other [FeCo] cluster complexes, the 532 nm light irradiation could also induce the transformation of both HS* → LS and LS → HS* owing to the partial overlap of the Co^II^ → Fe^III^ IVCT band and the broad Fe^II^ → Co^III^ IVCT band. Herein, the LS state of **1** was irradiated directly at 532 nm^40^,^41^ and the *χT* values reached a saturation value of 2.39 cm^3^ mol^–1^ K after 360 min (Fig. S10a, ESI†). The temperature-dependent *χT* values measured from 1.8 to 120 K show a maximum of 3.32 cm^3^ mol^–1^ K at 5 K (Fig. 2a). The ac magnetic susceptibilities displayed poor frequency dependence behavior and weak *χ*′ and *χ*′′ signals as well (Fig. S10b, ESI†). Therefore, the 532 nm laser irradiation had effects on both transformable directions.

The repeatability of the photo-switchable SCM behavior was demonstrated by tracing the time-dependent magnetic susceptibilities under successive and alternating irradiation at 808 and 532 nm (Fig. 2b). Furthermore, the temperature-dependent ac magnetic susceptibilities were measured in the third cycle. The ac signals are well repeatable (Fig. S11, ESI†), confirming the photo-reversible SCM behavior. We also performed the magnetization decay of the photo-induced HS* state at a series of temperatures (Fig. S12, ESI†) to see the stability. At high-temperatures (30–50 K), the relaxation time was strongly dependent on temperature, and in the temperature region of 10–20 K, it exhibited a weak temperature-dependence, suggesting that the relaxation from the metastable FeIIILSCoIIHSFeIIILS to the stable FeIILSCoIIILSFeIIILS was dominated by a quantum tunnelling mechanism in the low temperature region. Then, the effect of 532 nm irradiation on the metastable HS* state was compared with the natural relaxation cases at several temperatures. As shown in Fig. S13,† the *χT* values under irradiation at 532 nm decreased much faster than the nonirradiated ones at 10 K, 15 K and 20 K, suggesting that the 532 nm laser irradiation has a pronounced effect on the transformation from the metastable FeIIILSCoIIHSFeIIILS to the stable FeIILSCoIIILSFeIIILS. Above 30 K, this difference became smaller and almost disappeared at 50 K, indicating that the thermally induced relaxation is more predominant than the 532 nm irradiation. Thus, we have verified that complex **1** possessed the unique nature of the on/off-switching SCMs controlled by bidirectional photoinduced charge transfer at the given temperature.

### IR spectra analysis

The infrared (IR) spectra of **1** at 200 K displayed *v*_CN_ absorptions of 2073 and 2085 cm^–1^, characteristic of terminal groups of [FeIILS(bpy)(CN)_4_]^2–^. The absorption at 2122 cm^–1^ belonged to the terminal cyano groups of [FeIIILS(bpy)(CN)_4_]^–^. The *ν*_CN_ absorptions at 2192 and 2204 cm^–1^ were attributed to the bridging cyano groups of the FeIILS(μ-CN)CoIIILS and FeIIILS(μ-CN)CoIIILS linkages, respectively. The *ν*_CN_ stretches supported the assignment of the LS state in **1** (Fig. S14, ESI†).^17^,^35^

In addition, the IR spectra were recorded after irradiation by the 808 and 532 nm lasers at 10 K to provide further evidence for the photo-reversibility in **1** (Fig. S15, ESI†). After irradiating at 808 nm, the intensities of the bridging *ν*_CN_ absorptions (2192 and 2204 cm^–1^) decreased, while a new band appeared at 2173 cm^–1^, indicating that part of the FeIILS(μ-CN)CoIIILS linkages were photochemically transformed into FeIIILS(μ-CN)CoIIHS species.^17^,^35^ The new *ν*_CN_ absorption at 2105 cm^–1^ was typical of the intermediate state of the bridging FeIILS(μ-CN)CoIIHS.^54^ Then, a 532 nm laser was used to irradiate the metastable state, and the intensity of the *ν*_CN_ absorption for the FeIILS(μ-CN)CoIIILS linkage increased whereas that for the FeIIILS(μ-CN)CoIIHS decreased, suggesting that transformation from FeIIILS(μ-CN)CoIIHS to FeIILS(μ-CN)CoIIILS occurred *via* 532 nm laser irradiation. The intensity of the additional *ν*_CN_ absorption for the FeIILS(μ-CN)CoIIHS state decreased relatively. The optical study visually displayed that the bidirectional photo-response occurred in **1** at different wavelengths. In agreement with the cyanide-bridged [FeCo] analogues,^40^,^41^ the LS FeIILS–CoIIILS pairs and the photo-induced HS* FeIIILS–CoIIHS pairs in the 1D chain were sensitive to red and green light, respectively, in the region where the Fe^II^ → Co^III^ and Co^II^ → Fe^III^ IVCT bands are usually observed. Importantly, the bidirectionally photo-induced charge transfer processes were coupled with the concomitant change of anisotropy at the Co sites and intrachain magnetic coupling interactions between cyanide-bridged Fe and Co centers that are the crucial factors for the magnetic dipole alignment. Therefore, in contrast to the reported systems in which the light-driven SCM behavior could merely relax back on heating, the excitation and deexcitation of the SCM behavior studied here could be switched bidirectionally by two light sources at a given temperature based on the MMCT mechanism. The magnetic bistable states switched under light irradiations in either the present system or the light-actuated SMM^55^ provide the possibility of using molecular nanomagnetic materials for the read-in and erasure of high density computer memory or storage devices, which is directly analogous to current binary storage systems.

## Conclusions

In summary, the bidirectional photo-switchable SCM was successfully realized in the well isolated Fe(μ-CN)Co 1D chain for the first time, in which the on-switching SCM behavior driven by laser irradiation at 808 nm can be reversibly switched off by irradiation at 532 nm. The electronic structure evolution upon heating and light irradiations was well demonstrated and confirmed the reversible photo-switchable SCM behavior. This result has shown that the spin topology of SCMs can also be reversibly tuned *via* the photo-induced MMCT process, enabling access to turn on/off the SCM behavior with the potential application as high-density information storage materials for writing and erasing information upon light irradiations.

## Conflicts of interest

The authors declare no competing financial interests.

## Supplementary Material

Supplementary information

Crystal structure data
