# Supplementary material for: Switching single chain magnet behavior via photoinduced bidirectional metal-to-metal charge transfer
Source: Chem Sci. 2017 Oct 30;9(3):617–22. doi: 10.1039/c7sc03401f (PMC5868298; doi:10.1039/c7sc03401f)
Supplement: Supplementary file 1 [file SC-009-C7SC03401F-s001.pdf]

Electronic Supplementary Material (ESI) for Chemical Science.
This journal is © The Royal Society of Chemistry 2017

Electronic Supplementary Information

Switching Single Chain Magnet Behavior via Photoinduced Bidirectional Metal-to-Metal Charge Transfer

Wenjing Jiang, Chengqi Jiao, Yinshan Meng, Liang Zhao, Qiang Liu, Tao Liu*

State Key Laboratory of Fine Chemicals, Dalian University of Technology, 2 Linggong Rd., 116024, Dalian, China

*To whom correspondence should be addressed.

E-mail: liutao@dlut.edu.cn

Contents:

Experimental Section

Table S1 Crystal data and structure refinements for complex **1**.

Table S2 Selected bond lengths (Å) and angles (°) for complex **1** at 220 K.

Fig. S1 ^{57}Fe Mössbauer spectra of **1** at 78 K.

Fig. S2 (a) Temperature-dependent magnetic susceptibility of **1**. (b) Isothermal magnetization of **1** at 1.8 K. (c) Temperature dependence of the in-phase and out-of-phase components of ac magnetic susceptibility in a zero dc field at various ac frequencies and with a 3.5 Oe ac field.

Fig. S3 Solid state UV-vis spectra for **1** at 298 K.

Fig. S4 Plots of χT vs time for **1** irradiated under 808 nm and the metastable HS* state irradiated at 532, 405, 473, 671 nm at 10 K.

Fig. S5 Isothermal magnetization of **1** before irradiation, after irradiated at 808 nm and the metastable state irradiated at 532 nm at 1.8 K.

Fig. S6 Temperature dependence of the in-phase and out-of-phase components of ac magnetic susceptibility after irradiation at 808 nm (a) and 532 nm (b) in a zero dc field at various ac frequencies and with a 3.5 Oe ac field.

Fig. S7 Arrhenius plots for the magnetic relaxation process of **1** after irradiation at 808 nm based on the peak values of χ''.

Fig. S8 Cole-Cole diagram for **1** after irradiation at 808 nm at 2.7 K. The solid red line represents the best fit of the experimental results with a generalized Debye model.

Fig. S9 (a) Plot of ln($\chi'T$) versus 1/T for **1** after irradiation at 808 nm. (b) Plot of ln($\chi'T$) versus 1/T for the metastable HS* state of **1** after irradiation at 532 nm (χ' is the in-phase component of the ac susceptibility measured in zero dc field at 1 Hz).

Fig. S10 (a) Plots of χT vs time for **1** irradiated under 532 nm at 10 K. (b) Temperature dependence of the in-phase and out-of-phase components of ac magnetic susceptibility of **1** in a zero dc field at various ac frequencies and with a 3.5 Oe ac field after irradiation at 532 nm .

Fig. S11 Temperature dependence of the in-phase and out-of-phase components of ac magnetic susceptibility after irradiation at 808 nm (a) and 532 nm (b) in a zero dc field at various ac frequencies and with a 3.5 Oe ac field in the third cycle.

Fig. S12 (a) Relaxation kinetics of the photoinduced fraction in normalized values (t = 0, converted fraction = 1) of **1** at 10 K (green), 15 K (blue), 20 K (red), 30 K (orange), 40 K (violet), 50 K (cyan) in the dark. The black solid lines are the stretched-exponential fit to Equation: $\gamma(t) = \gamma(0)\exp(-t/\tau)^{\beta}$. (b) Corresponding Arrhenius plots were shown with parameters describing the relaxation kinetics of **1** in the high-temperature range (30–50 K) and low-temperature range (10–20 K).

Fig. S13 Plots of χT *vs* time for the metastable HS* state of **1** irradiated at 532 nm and the relative thermal-relaxation at 10, 15, 20, 30, 40, 50 K.

Fig. S14 Solid state FT-IR spectra for **1** at 200 K.

Fig. S15 The IR spectra for **1** irradiated at 808 nm and the photo-reversibility upon irradiation at 532 nm at 10 K.

Experimental Section

Materials

All chemical reagents were commercially available and used without further purification. Li[Fe(papy)$(CN)_4$] (papy = 2,2′-dipyridine) were synthesized according to the literature method.[1]

References

1 R. Lescouëzec, F. Lloret, M. Julve, J. Vaissermann, M. Verdaguer, R. Llusar, S. Uriel, *Inorg. Chem.*, 2001, **40**, 2065−2072.

Synthesis of Complex 1

Complex **1** was synthesized by a diffusion method in a test tube. A mixture of methanol/water (1:4, *v*/*v*, 3 mL) was gently layered on the top of a solution of $Co(ClO_4)_2{\cdot}6H_2O$ (0.0050 mmol) in water (1.0 mL), and then a 1.0 mL methanol solution of Li[Fe(bipy)$(CN)_4$]$\cdot H_2O$ (0.010 mmol) and 4-phenylpyridine (0.010 mmol) was carefully added as the third layer. After a few weeks, red flake crystals of **1** were collected, washed with water and air dried. Yield: 52% based on $Co(ClO_4)_2{\cdot}6H_2O$. Anal. calcd (%) for $Fe_2CoC_{50}H_{38}N_{14}O_2$: C 57.88, H 3.69, N 18.90; found: C 57.91, H 3.65, N 18.92.

Structure Determination and Refinement

The single-crystal XRD data for **1** were collected on a Bruker Smart APEX II X-diffractometer equipped with graphite monochromated Mo-Kα radiation (λ = 0.71073 Å) using the SMART and SAINT programs at 220 K. Final unit cell parameters were based on all observed reflections from integration of all frame data. The structures were solved in the space group by direct method and refined by the full-matrix least-squares using SHELXTL-97 fitting on F^2. For all compounds, all non-hydrogen atoms were refined anisotropically. The hydrogen atoms of organic ligands were located geometrically and fixed isotropic thermal parameters.

IR and UV-Vis Spectra measurements

Infrared spectra were measured on KBr pellets samples using a Nicolet IS10 FT-IR spectrometer equipped with a liquid helium type cryostat (Optistat CF_2). For infrared spectra after irradiation, the sample was continuously irradiated via a flexible optical fiber guided laser diode pumped Nd:YAG laser (λ = 808 nm, 39 mW/cm^2) and Nd:YAG laser (λ = 532 nm， 10 mW/cm^2) at 10 K. Solid state UV-Vis absorption spectra were recorded on a HITACHIU-4100 UV-Vis spectrophotometer.

Magnetic and Photomagnetic Studies

Magnetic measurements were performed on a Quantum Design SQUID (MPMSXL-7) magnetometer with the polycrystalline samples. To prevent the loss of uncoordinated solvents, the fresh sample was loaded directly into the sample chamber at 110K (He atmosphere) and frozen before purging under vacuum. Variable-temperature magnetic measurements of the heating mode ware carried out from 2 K to 300 K. Data were corrected for the diamagnetic contribution calculated from Pascal constants.

Photomagnetic measurements were carried out with the same instrument but by using a sample equipped with an optical fiber. The powder sample of **1** was deposited on a commercial adhesive tape and placed on the edge of the optical fiber. The photo-irradiation was performed with a laser diode pumped Nd:YAG lasers (λ = 808 nm, 39 mW/cm^2; λ = 532 nm, 10 mW/cm^2; λ = 473 nm, 10 mW/cm^2; λ = 405 nm, 5 mW/cm^2) and Nd: YVO4 laser (λ = 671 nm, 12 mW/cm^2). The temperature-dependent magnetization was measured both before and after irradiation in the range of 1.8–120 K. The difference in the magnetization before and after irradiation was extracted by subtracting the magnetization value before irradiation from that after irradiation.

Table S1 Crystal data and structure refinements for complex **1**.

Temperature, K	**220**
CCDC	1528877
formula	$Fe_2CoC_{50}H_{38}N_{14}O_2$
Fw	1037.55
crystal system	Monoclinic
Space group	$C_{2/c}$
a, Å	27.352(12)
b, Å	15.144(7)
c, Å	13.336(6)
α, °	90
β, °	116.222(7)
γ, °	90
V, Å^3	4956(4)
Z	4
D_c, g/cm^3	1.364
μ (Mo K_α), mm^{-1}	0.963
crystal size, mm^3	0.08×0.05×0.04
θ_{min}, θ_{max}, °	2.578, 27.533
no. total reflns.	15451
no. uniq. reflns. (R_{int})	5571 (0.0562)
no. params.	313
R_1, wR_2 [I≥2σ(I)]	0.0496, 0.1533
R_1, wR_2 (all data)	0.0763, 0.1695
GOF	1.070

Table S2 Selected bond lengths (Å) and angles (°) for complex **1** at 220 K.

Fe(1)-C(13)	1.882(3)	Fe(1)-C(12)	1.886(3)
Fe(1)-C(14)	1.950(4)	Fe(1)-C(11)	1.955(4)
Fe(1)-N(1)	1.981(3)	Fe(1)-N(2)	1.985(3)
Co(1)-N(4)#1	1.885(3)	Co(1)-N(4)#2	1.885(3)
Co(1)-N(3)#3	1.892(3)	Co(1)-N(3)	1.892(3)
Co(1)-N(7)	1.975(3)	Co(1)-N(7)#3	1.975(3)
N(4)-Co(1)#2	1.885(3)		
C(13)-Fe(1)-C(12)	86.24(14)	C(13)-Fe(1)-C(14)	92.72(14)
C(12)-Fe(1)-C(14)	86.99(14)	C(13)-Fe(1)-C(11)	88.67(14)
C(12)-Fe(1)-C(11)	89.89(14)	C(14)-Fe(1)-C(11)	176.49(14)
C(13)-Fe(1)-N(1)	96.36(13)	C(12)-Fe(1)-N(1)	176.70(12)
C(14)-Fe(1)-N(1)	90.85(13)	C(11)-Fe(1)-N(1)	92.20(13)
C(13)-Fe(1)-N(2)	176.75(12)	C(12)-Fe(1)-N(2)	96.46(13)
C(14)-Fe(1)-N(2)	89.24(13)	C(11)-Fe(1)-N(2)	89.52(13)
N(1)-Fe(1)-N(2)	81.01(12)	N(4)#1-Co(1)-N(4)#2	180.0(3)
N(4)#1-Co(1)-N(3)#3	91.12(12)	N(4)#2-Co(1)-N(3)#3	88.88(12)
N(4)#1-Co(1)-N(3)	88.88(12)	N(4)#2-Co(1)-N(3)	91.12(12)
N(3)#3-Co(1)-N(3)	180.0	N(4)#1-Co(1)-N(7)	89.59(12)
N(4)#2-Co(1)-N(7)	90.41(12)	N(3)#3-Co(1)-N(7)	92.32(11)
N(3)-Co(1)-N(7)	87.68(11)	N(4)#1-Co(1)-N(7)#3	90.42(12)
N(4)#2-Co(1)-N(7)#3	89.58(12)	N(3)#3-Co(1)-N(7)#3	87.68(11)
N(3)-Co(1)-N(7)#3	92.32(11)	N(7)-Co(1)-N(7)#3	180.0
C(16)-N(7)-Co(1)	121.9(2)	C(15)-N(7)-Co(1)	119.5(2)
C(10)-N(2)-Fe(1)	126.5(2)	C(6)-N(2)-Fe(1)	114.9(2)
C(12)-N(3)-Co(1)	164.2(3)	N(3)-C(12)-Fe(1)	179.0(3)
C(5)-N(1)-Fe(1)	115.2(2)	C(1)-N(1)-Fe(1)	126.2(2)
N(6)-C(11)-Fe(1)	178.8(4)	N(4)-C(13)-Fe(1)	175.2(3)
N(5)-C(14)-Fe(1)	177.7(3)	C(13)-N(4)-Co(1)#2	172.6(3)

Symmetry transformations used to generate equivalent atoms:

#1 x, –y, z+1/2; #2 –x, y, –z–1/2; #3 –x, –y, –z.

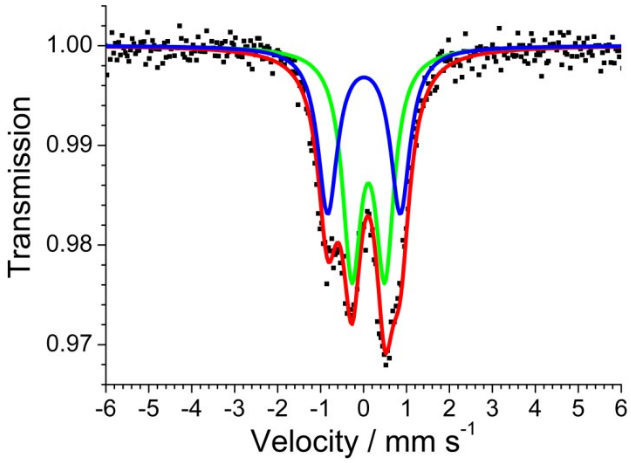

Fig. S1 [57]Fe Mössbauer spectra of **1** at 78 K. Mössbauer parameters: $\delta = -0.03$ and $\Delta E_Q = 1.69$ mm s^{-1}, corresponding to LS Fe^{III}; $\delta = 0.07$ and $\Delta E_Q = 0.77$ mm s^{-1}, corresponding to the LS Fe^{II}. The Fe^{III}/Fe^{II} peak area ratio was 0.51/0.49, suggesting the LS state with the $Fe^{III}_{LS}(\mu\text{-CN})Co^{III}_{LS}(\mu\text{-NC})Fe^{II}_{LS}$ linkages. The solid lines are Loretzian curves plotted by the calculated values.

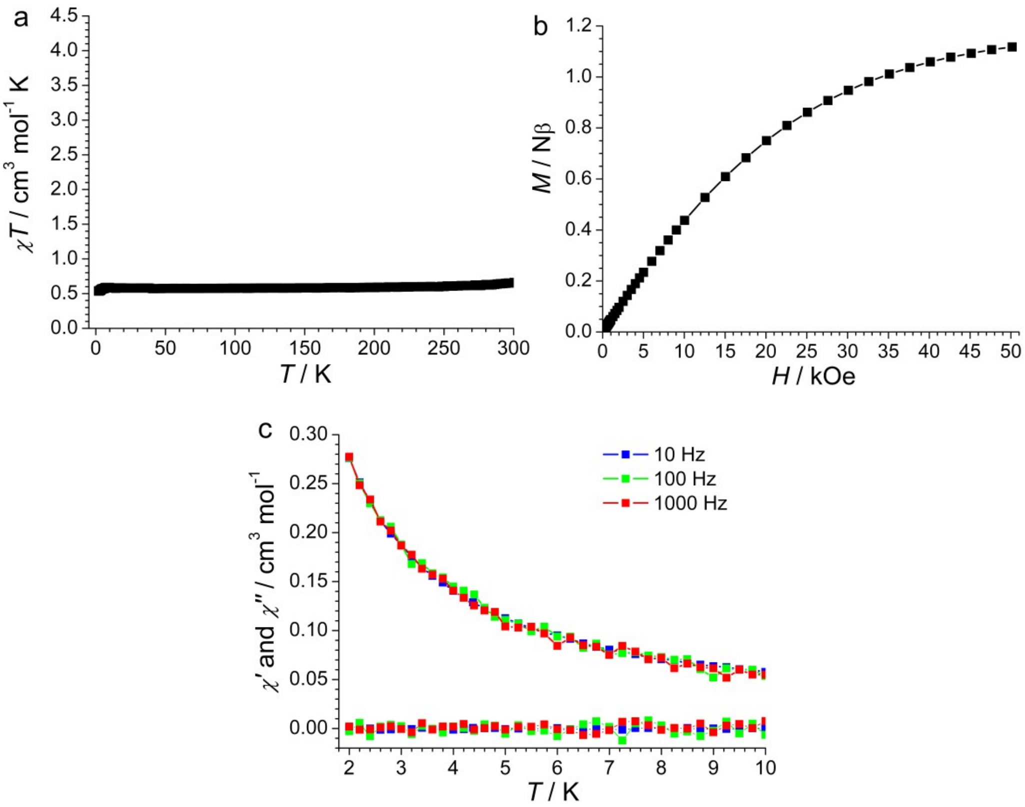

Fig. S2 (a) Temperature-dependent magnetic susceptibilities of **1** under a dc field of 1000 Oe. (b) Isothermal magnetization of **1** at 1.8 K. (c) Temperature dependence of the in-phase and out-of-phase components of ac magnetic susceptibility in a zero dc field at various ac frequencies and with a 3.5 Oe ac field.

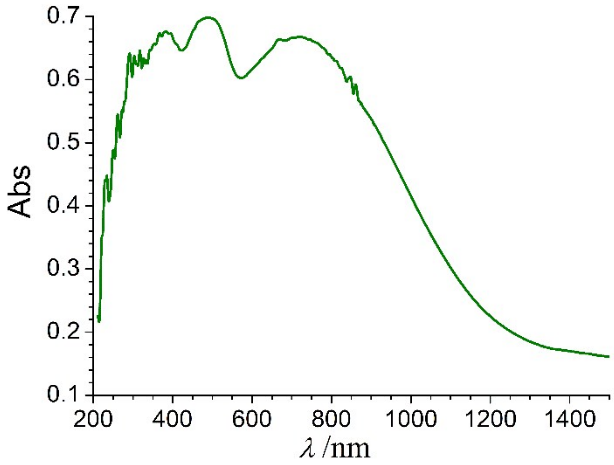

Fig. S3 Solid state UV-vis spectra for **1** at 298 K.

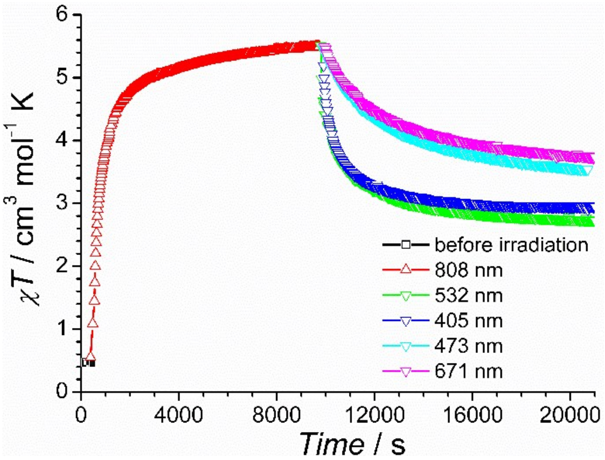

Fig. S4 Plots of χT vs time for **1** irradiated under 808 nm and the metastable HS* state irradiated at 532, 405, 473, 671 nm at 10 K.

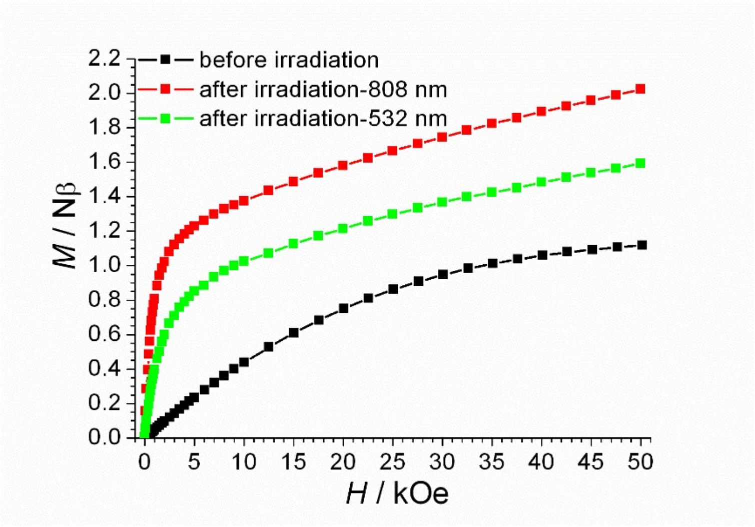

Fig. S5 Isothermal magnetization of **1** before irradiation, after irradiated at 808 nm and the metastable state irradiated at 532 nm at 1.8 K.

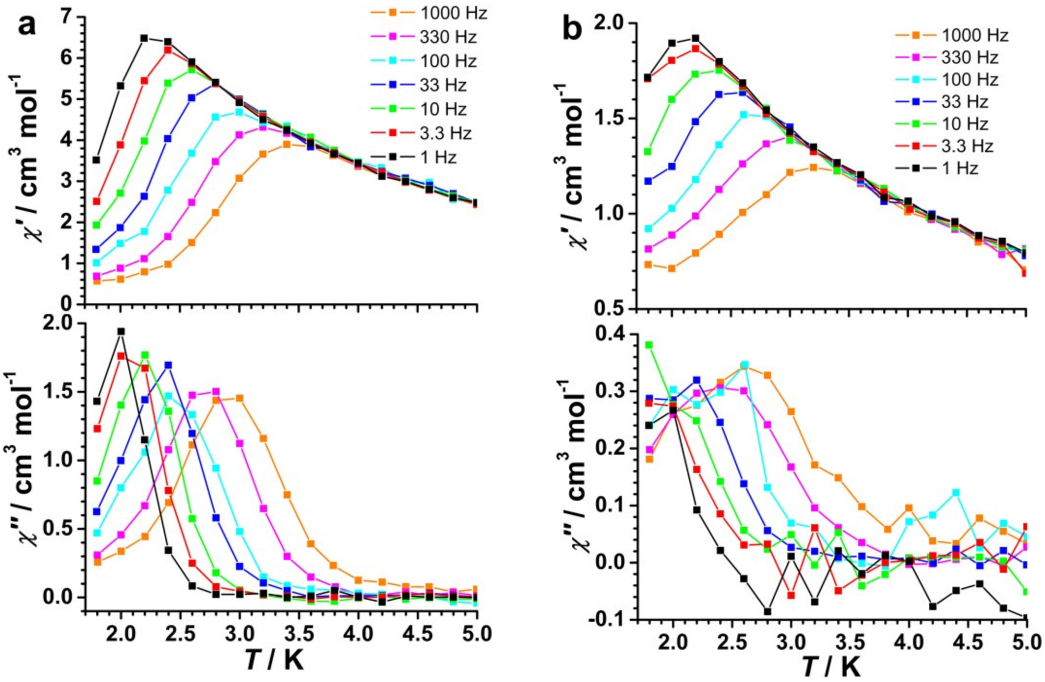

Fig. S6 Temperature dependence of the in-phase and out-of-phase components of ac magnetic susceptibility of **1** after irradiation at 808 nm (a) and 532 nm (b) in a zero dc field at various ac frequencies and with a 3.5 Oe ac field.

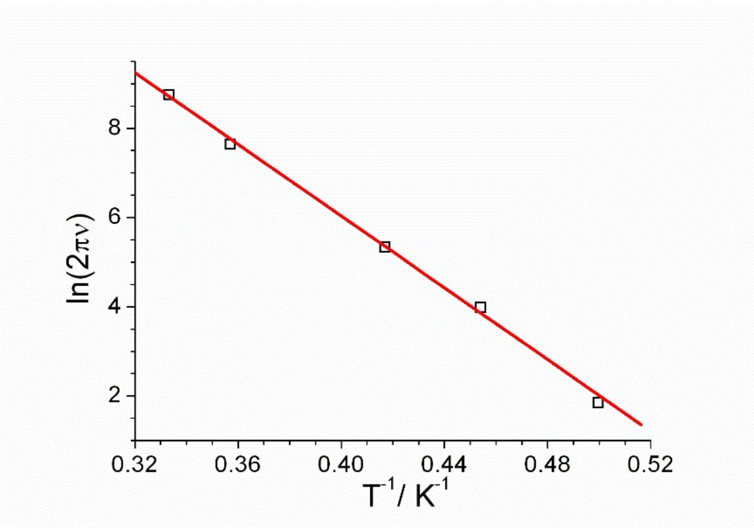

Fig. S7 Arrhenius plots for the magnetic relaxation process of **1** after irradiation at 808 nm based on the peak values of χ''.

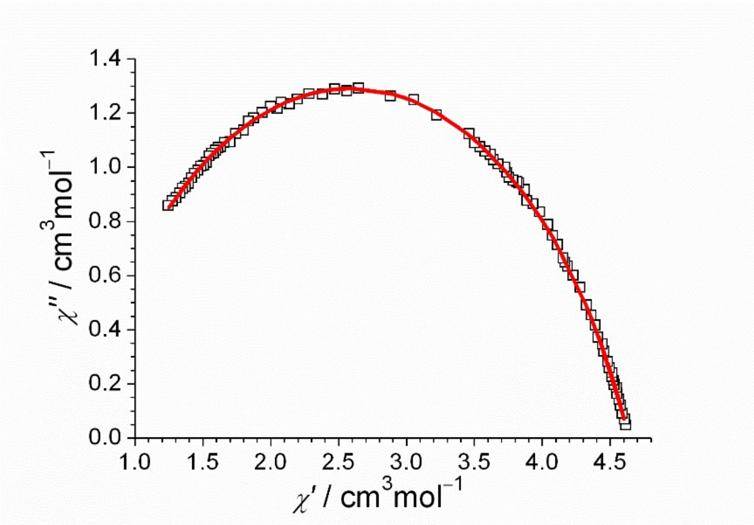

Fig. S8 Cole-Cole diagram for **1** after irradiation at 808 nm at 2.7 K. The solid red line represents the best fit of the experimental results with a generalized Debye model.

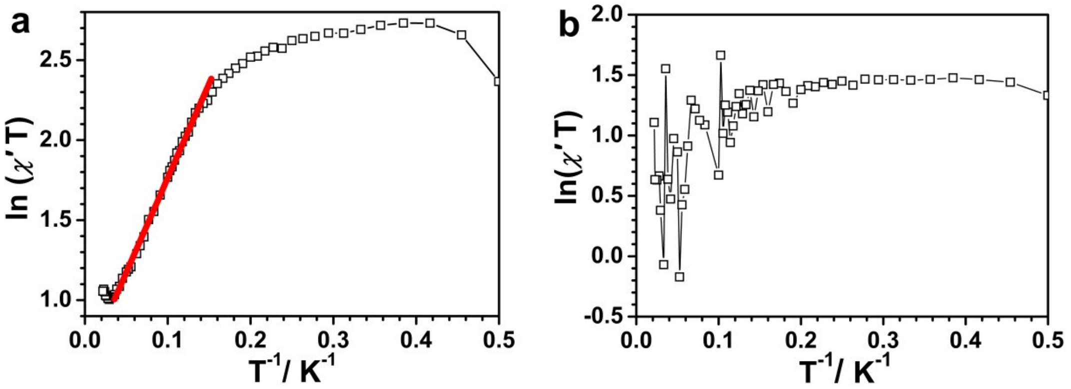

Fig. S9 (a) Plot of ln($\chi'T$) versus $1/T$ for **1** after irradiation at 808 nm. (b) Plot of ln($\chi'T$) versus $1/T$ for the metastable HS* state of **1** after irradiation at 532 nm (χ' is the in-phase component of the ac susceptibility measured in zero dc field at 1 Hz).

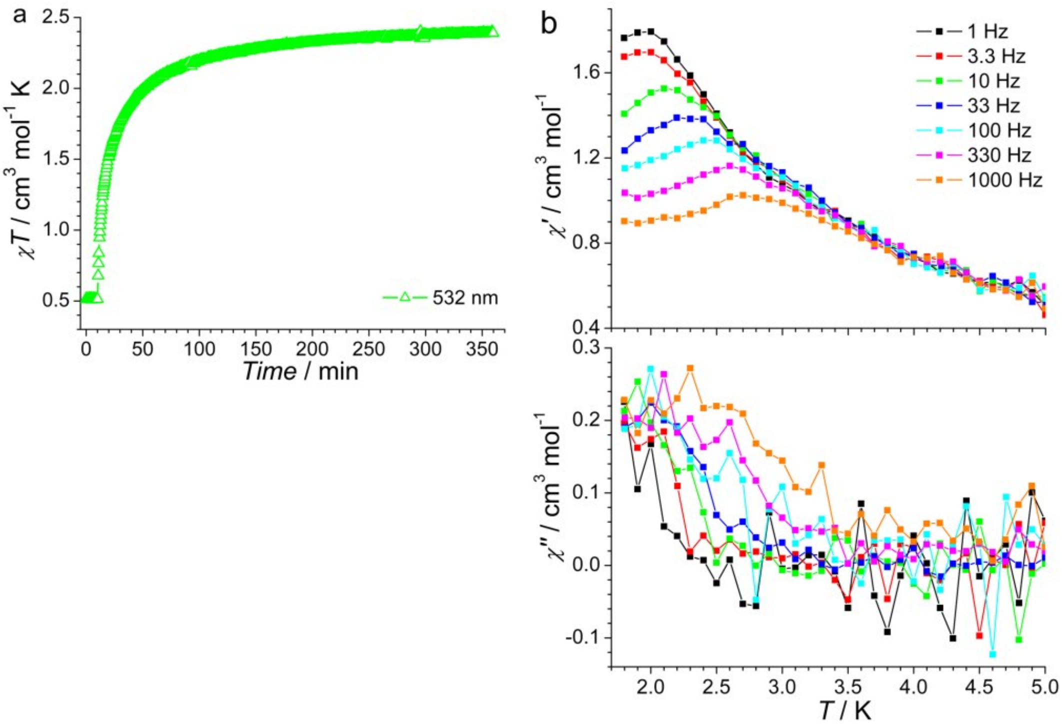

Fig. S10 (a) Plots of χT vs time for **1** irradiated under 532 nm at 10 K. (b) Temperature dependence of the in-phase and out-of-phase components of ac magnetic susceptibility of **1** in a zero dc field at various ac frequencies and with a 3.5 Oe ac field after irradiation at 532 nm .

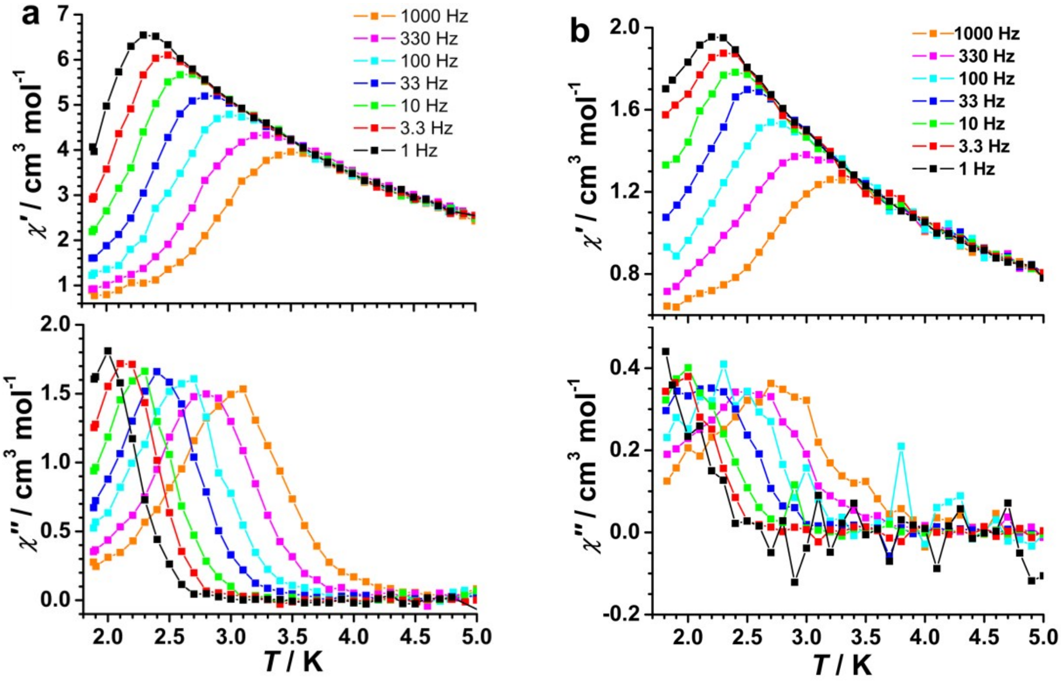

Fig. S11 Temperature dependence of the in-phase and out-of-phase components of ac magnetic susceptibility of **1** after irradiation at 808 nm (a) and 532 nm (b) in a zero dc field at various ac frequencies and with a 3.5 Oe ac field in the third cycle.

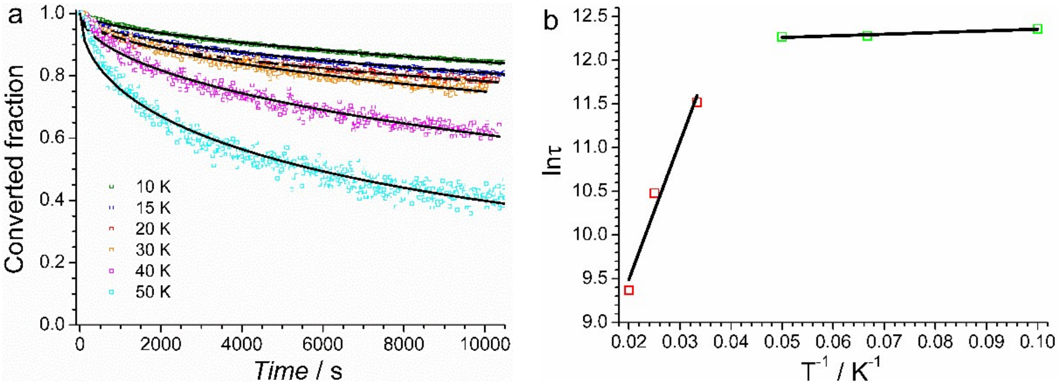

Fig. S12 (a) Relaxation kinetics of the photoinduced fraction in normalized values (t = 0, converted fraction = 1) of **1** at 10 K (green), 15 K (blue), 20 K (red), 30 K (orange), 40 K (violet), 50 K (cyan) in the dark. The black solid lines are the stretched-exponential fit with Equation: $\gamma(t) = \gamma(0)\exp(-t/\tau)^{\beta}$. (b) Corresponding Arrhenius plots were shown with parameters describing the relaxation kinetics of **1** in the high-temperature range (30–50 K) and low-temperature range (10–20 K).

Relaxation of the photoinduced metastable state was monitored at different temperatures to probe the stability of the photoinduced phases. The decay of magnetization was normalized to the photoinduced fraction γ and fitted to a stretched exponential law:

$\gamma(t) = \gamma(0)\exp(-t/\tau)^{\beta}$,

where τ represents the relaxation time of the system at temperature T and β represents the factor of time-evolving magnetic interactions during the decay owing to different magnetic interaction topologies in the high- and low-temperature phases (Fig. S12a). The obtained relaxation time τ was fitted to an Arrhenius relationship ($\tau(T) = \tau_0\exp(\Delta E/(k_B T))$, where τ_0 and $\Delta E/k_B$ represent the pre-exponential factor and the energy barrier, respectively. Two distinct dependencies of τ on temperature were observed (Fig. S12b). In the low-temperature (10–20 K) region, τ was less dependent on temperature. A best fitting procedure gave $\Delta E/k_B$ = 1.9(±0.4) cm^{-1} and $\tau_0 = 2.0\times10^5$ s. In the high-temperature range (30–50 K), the relaxation time τ was strongly dependent on temperature, giving an energy barrier $\Delta E/k_B$ = 157.5(±25.7) cm^{-1} and τ_0 = 568.7 s.

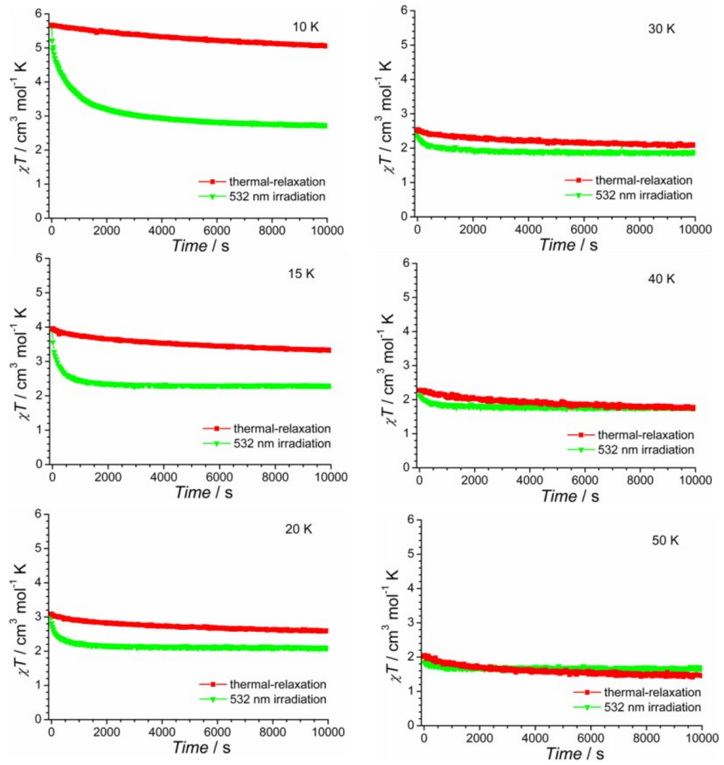

Fig. S13 Plots of χT vs time for the metastable HS* state of **1** irradiated at 532 nm and the relative thermal-relaxation at 10, 15, 20, 30, 40, 50K.

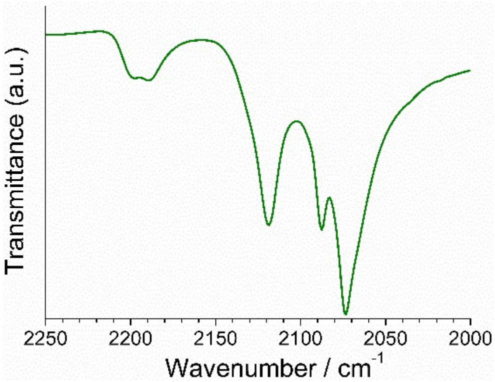

Fig. S14 Solid state FT-IR spectra for **1** at 200 K.

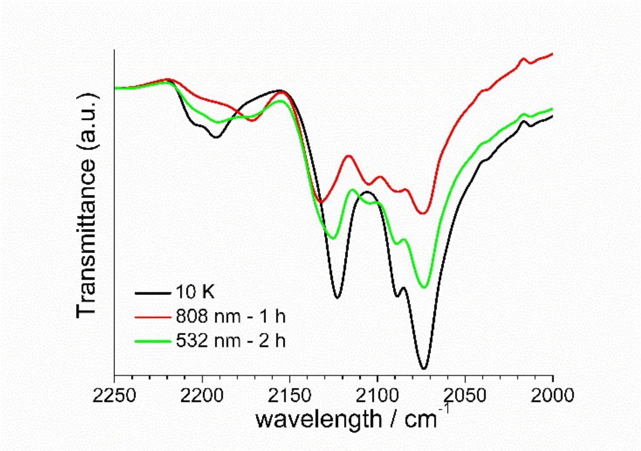

Fig. S15 The IR spectra for **1** irradiated at 808 nm and the photo-reversibility upon irradiation at 532 nm at 10 K.
